# The Effect of COVID-19 Lockdown on Cerebrovascular Accidents, Acute Coronary Syndrome, and Diabetic Ketoacidosis Visits the Emergency Department: A Retrospective Study

**DOI:** 10.7759/cureus.33154

**Published:** 2022-12-30

**Authors:** Abdullah A Ayoub, Malik J Addas, Abdullah A Alghamdi, Nada Alghazzawi, Abdullah Bakhsh, Turki Alharbi

**Affiliations:** 1 College of Medicine, King Abdulaziz University Faculty of Medicine, Jeddah, SAU; 2 Department of Emergency Medicine, King Abdulaziz University Faculty of Medicine, Jeddah, SAU

**Keywords:** cerebrovascular accidents (cva), acute coronary syndrome (acs), stroke, lockdown, covid-19, visit rates, emergency department, pandemic emergency medicine, diabetic ketoacidosis (dka)

## Abstract

Objective

This study aims to assess the effect of the COVID-19 lockdown on acute coronary syndrome (ACS), cerebrovascular accident (CVA), and diabetic ketoacidosis (DKA) visits the emergency department (ED).

Methods

We compared two groups of patients attending King Abdulaziz University Hospital (KAUH) ED diagnosed with one of the following ACS, CVA, or DKA; patients presenting from 21 December 2019 to 23 March 2020 and patients presenting from 23 March 2020 to 21 June 2020, representing COVID-19 pre-lockdown and during-lockdown period, respectively. The variables we analyzed were age, nationality (Saudi/non-Saudi), and gender.

Results

Our total sample size was 285 patient visits, ACS (n=130), CVA (n=98), and DKA (n=57). Results showed a statistically significant relationship between the number of patients with ACS and the period of visitation to ED (45.24% reduction, p-value <0.001), while CVA (18.5% reduction, p-value 0.312) and DKA (16% reduction, p-value 0.508) showed no statistically significant relationship.

Conclusion

A lockdown may be necessary to control a pandemic. However, it may carry potential collateral damage, such as a decrease and delay in the presentation of life-threatening conditions, which may lead to worsening outcomes.

A clinical presentation of these conditions should warrant comprehensive evaluation by healthcare workers regardless of an ongoing pandemic while implementing infection control guidelines.

## Introduction

In late December 2019, a new strand of highly contagious virus spread through respiratory droplets emerged from Wuhan in China. It was identified as severe acute respiratory syndrome coronavirus 2 (SARS-CoV-2), which is the cause of coronavirus disease 2019 (COVID-19). Presentation varied from asymptomatic to acute respiratory distress syndrome and, ultimately, death.

Because of the rapid outbreak of the virus worldwide, the World Health Organization (WHO) declared a worldwide pandemic on 11 March 2020; in response, countries worldwide initiated flight bans and lockdowns [1].

The first confirmed case in Saudi Arabia was reported on 2 March 2020. Then on 15 March, all international flights to and from Saudi Arabia were suspended, and on 23 March, a national lockdown was declared [1].

Every year 17.9 million people die from cardiovascular diseases; myocardial infarction (MI) and cerebrovascular accident (CVA) represent 85% of these deaths [2].

MI develops when a part of the heart's wall gets ischemic from lack of oxygen supply due to atherosclerotic plaque narrowing the blood vessel supplying it. In the United States, 500,000-700,000 people die every year from MI, one every 40 seconds [3], while in Saudi Arabia, MI patients represent 5.5% of the population [4].

A sudden loss of blood supply to an area of the brain causes CVA, which can be due to ischemic (e.g., atherosclerosis) or hemorrhagic (e.g., aneurysm rupture) causes. According to WHO, 15 million people experience a stroke yearly [5].

Diabetic ketoacidosis (DKA) results from uncontrolled high blood sugar, mainly in diabetes mellitus type 1. DKA's worldwide mortality rate dropped from 8% to 1% over the years. Two studies in Saudi Arabia reported 2.9% and 3.5% mortality rates [6].

The United States Centers for Disease Control and Prevention (CDC) Morbidity and Mortality Weekly Report (MMWR) also identified an immediate decrease of 42% in emergency department (ED) visits after the declaration of COVID-19 as a public health emergency from Jan-May 2020; ED visits declined 23% for MI, 20% for stroke, and 10% for a hyperglycemic crisis [7]. Another research in Saudi Arabia showed increased mortality in patients visiting the ED during lockdown with non-COVID-19 diagnoses due to presentation delay [8].

This study aims to assess the effect of the COVID-19 lockdown on acute coronary syndrome (ACS), CVA, and DKA visits to the ED.

## Materials and methods

Study design

This research is a retrospective study comparing two groups of patients attending King Abdulaziz University Hospital (KAUH) ED diagnosed with one of the following ACS, CVA, or DKA; patients presenting from 21 December 2019 to 23 March 2020 and patients presenting from 23 March 2020 to 21 June 2020, representing COVID-19 pre-lockdown and during-lockdown period, respectively.

Inclusion Criteria

We included all patients presenting to the KAUH ED within the COVID-19 pre-lockdown or during-lockdown periods and who were diagnosed with ACS, CVA, or DKA, regardless of past medical history.

Exclusion Criteria

Patients who were presenting outside of the COVID-19 pre-lockdown and during-lockdown periods and/or who were diagnosed with a disease other than ACS, CVA, or DKA.

The study proposal was approved by the King Abdulaziz University (KAU) Faculty of Medicine - Research Ethics Committee (REC) under Reference No 260-21 as a Non-Intervention (Retrospective Record Review) dated 19 April 2021. The number of registration at the National Committee of Bio. &Med. Ethics (HA-02-J-008).

Statistical analysis

The variables we analyzed were age, nationality (Saudi/non-Saudi), and gender, which we described descriptively.

Data were analyzed using IBM Corp. Released 2019. IBM SPSS Statistics for Windows, Version 26.0. Armonk, NY: IBM Corp. Qualitative data were expressed as numbers and percentages, and the Chi-squared test (χ2) was used to assess relationships between the categorical study variables. Quantitative data were expressed in terms of their mean and standard deviation (Mean ± SD). Independent samples t-test was used to compare the means of quantitative variables before and during the COVID-19 lockdown. Two sample proportion test was used to compare the proportions of disease diagnosis before and during the COVID-19 lockdown. A p-value of less than 0.05 was considered statistically significant.

## Results

Our total sample size was 285 patient visits. ACS (n=130), CVA (n=98), and DKA (n=57).

**Figure 1:**
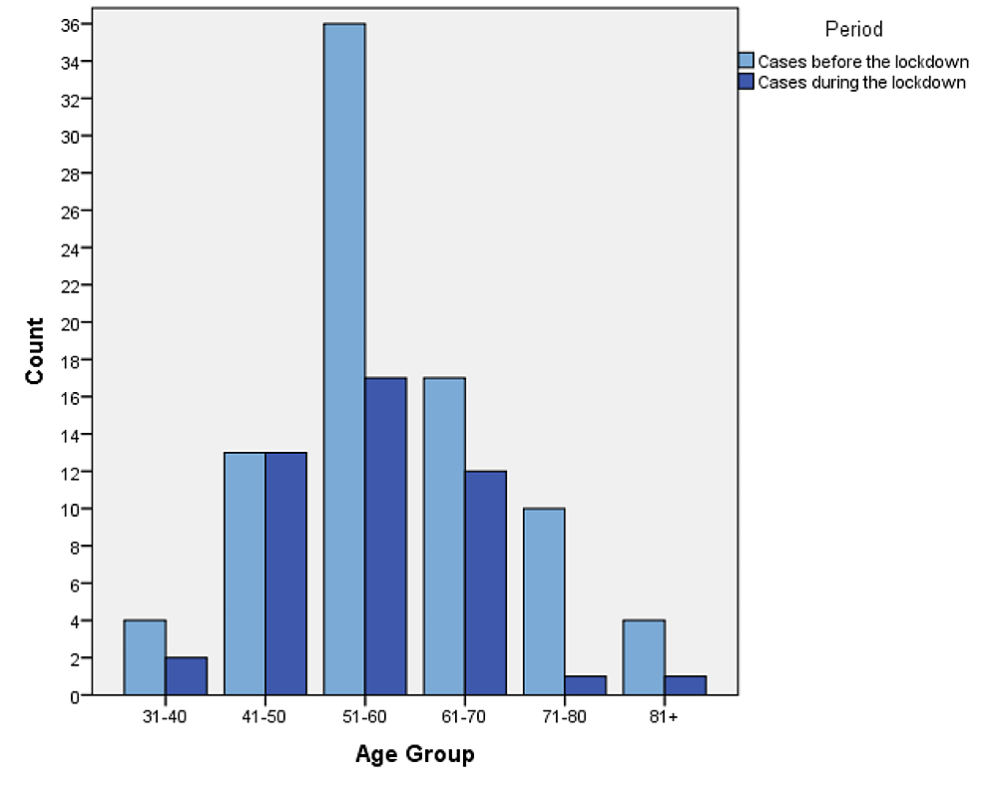
The number of ACS cases according to age groups before and during the lockdown.

**Figure 2:**
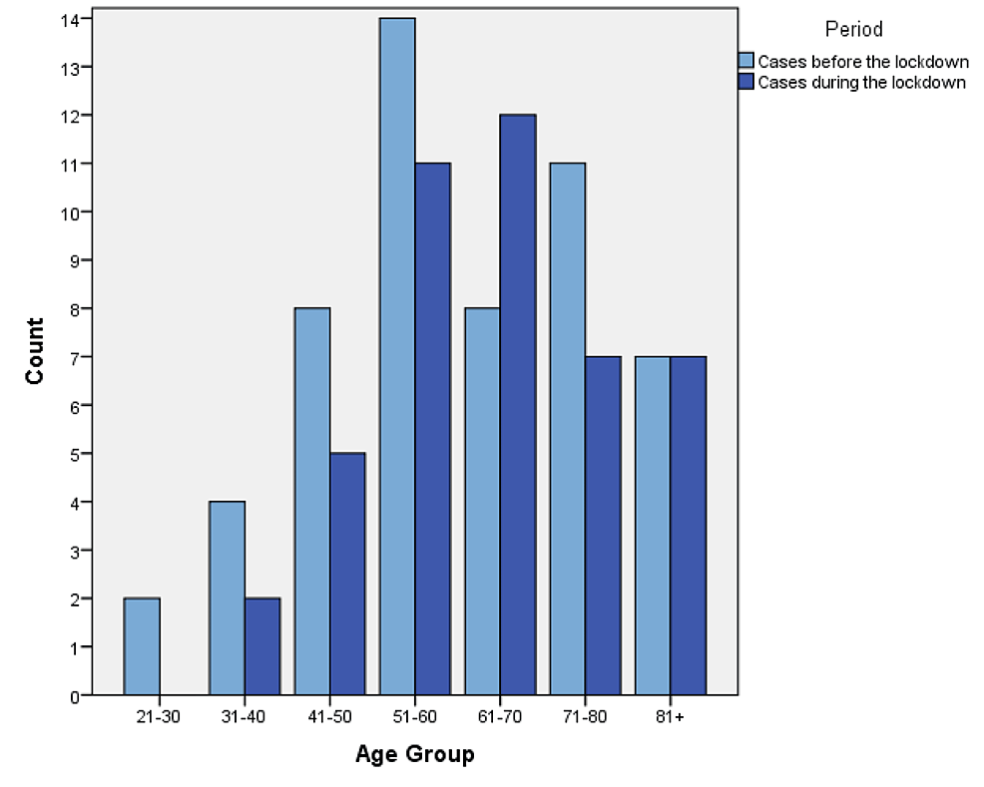
The number of CVA cases according to age groups before and during the lockdown.

**Figure 3:**
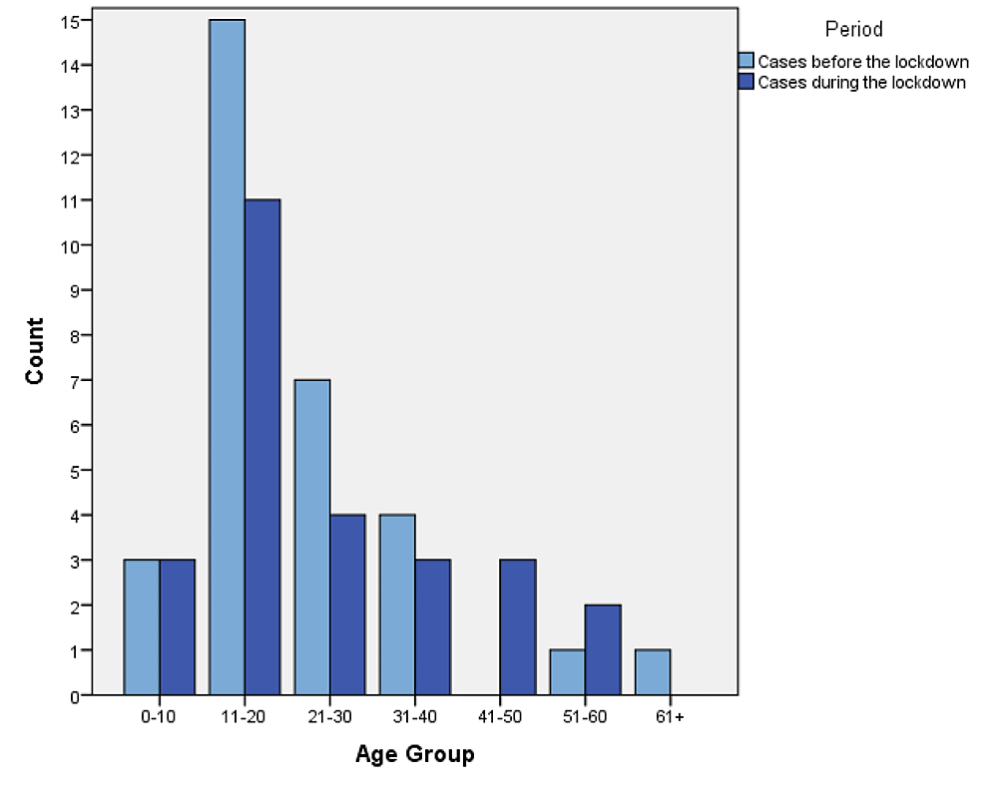
The number of DKA cases according to age groups before and during the lockdown.

We categorized data in terms of disease and period of presentation. A breakdown of cases according to gender and nationality is detailed in Table 1, while descriptive statistics of ages are shown in Table 2. The number of cases according to age groups before and during the lockdown is visually represented in Figures 1-3.

**Table 1:** Breakdown of cases according to gender and nationality, categorized in terms of disease and period of presentation.

		Gender		Nationality	
		Male	Female	Saudi	Non-Saudi
ACS	Cases before the lockdown	74	10	19	65
	Cases during the lockdown	40	6	9	37
	Total	114	16	28	102
CVA	Cases before the lockdown	33	21	18	36
	Cases during the lockdown	25	19	19	25
	Total	58	40	37	61
DKA	Cases before the lockdown	13	18	15	16
	Cases during the lockdown	11	15	18	8
	Total	24	33	33	24

**Table 2:** Descriptive statistics of ages, categorized in terms of disease and period of presentation.

		n	Minimum Age	Maximum Age	Mean Age	Std. Deviation
ACS	Cases before the lockdown	84	34	93	58.976	11.5257
	Cases during the lockdown	46	32	85	55.413	10.1227
CVA	Cases before the lockdown	54	25	90	61.222	15.7128
	Cases during the lockdown	44	38	97	64.977	14.8957
DKA	Cases before the lockdown	31	2	62	22.935	13.319
	Cases during the lockdown	26	6	58	24.115	15.3527

Results showed a statistically significant relationship between the number of patients with ACS and the period of visitation to the ED, as shown in Table 3. ACS showed a 45.24% reduction with a p-value <0.001, while CVA (18.5% reduction, p-value 0.312) and DKA (16% reduction, p-value 0.508) showed no statistically significant relationship. Analysis of independent variables showed no significant relationship, as shown in Table 4.

**Table 3:** Statistical analysis of ACS, CVA, and DKA in relation to lockdown periods. (1) Z-test for proportion.

	Period	Count	Proportion	χ²	df	p-value
ACS	Before the lockdown	84	0.646	11.1	1	<0.001^1^
	During the lockdown	46	0.354			
CVA	Before the lockdown	54	0.551	1.02	1	0.312^1^
	During the lockdown	44	0.449			
DKA	Before the lockdown	31	0.544	0.439	1	0.508^1^
	During the lockdown	26	0.456			

**Table 4:** Statistical analysis of independent variables in relation to lockdown periods. (1) Pearson's Chi-squared test.

			Before the lockdown	During the lockdown	Total	p-value
ACS	Gender	Female	10 (11.9)	6 (13.0)	16 (12.3)	0.999^1^
		Male	74 (88.1)	40 (87.0)	114 (87.7)	
	Age	<40	4 (4.8)	2 (4.3)	6 (4.6)	0.66^1^
		40-60	44 (52.4)	27 (58.7)	71 (54.6)	
		61-80	30 (35.7)	16 (34.8)	46 (35.4)	
		>80	6 (7.1)	1 (2.2)	7 (5.4)	
	Nationality	Non-Saudi	65 (77.4)	37 (80.4)	102 (78.5)	0.856^1^
		Saudi	19 (22.6)	9 (19.6)	28 (21.5)	
CVA	Gender	Female	21 (38.9)	19 (43.2)	40 (40.8)	0.823^1^
		Male	33 (61.1)	25 (56.8)	58 (59.2)	
	Age	<40	4 (7.4)	2 (4.5)	6 (6.1)	0.887^1^
		40-60	20 (37.0)	15 (34.1)	35 (35.7)	
		61-80	21 (38.9)	20 (45.5)	41 (41.8)	
		>80	9 (16.7)	7 (15.9)	16 (16.3)	
	Nationality	Non-Saudi	36 (66.7)	25 (56.8)	61 (62.2)	0.429^1^
		Saudi	18 (33.3)	19 (43.2)	37 (37.8)	
DKA	Gender	Female	18 (58.1)	15 (57.7)	33 (57.9)	0.999^1^
		Male	13 (41.9)	11 (42.3)	24 (42.1)	
	Age	<40	28 (90.3)	21 (80.8)	49 (86.0)	0.238^1^
		40-60	2 (6.5)	5 (19.2)	7 (12.3)	
		61-80	1 (3.2)	0 (0.0)	1 (1.8)	
	Nationality	Non-Saudi	16 (51.6)	8 (30.8)	24 (42.1)	0.187^1^
		Saudi	15 (48.4)	18 (69.2)	33 (57.9)	

## Discussion

The period of national lockdown declared in Saudi Arabia showed a reduction in the presentation of acute life-threatening conditions to the ED compared to the period before the lockdown.

The reduction in visitations could be attributed to public fear of catching the virus and the government's reaction to the virus by forcing a national lockdown, making it difficult to seek medical attention. Plus, hospitals' primary focus was fighting the pandemic, thus minimizing care for other conditions. In addition to the lack of national education and campaigns to not neglect the red flags of these critical illnesses and the need to visit EDs.

ACS showed the most reduction in visitations (45.24% reduction, p-value <0.001). The mean age of patients who presented with ACS during our study period was 58 and 55 years old before and during the lockdown, respectively. Knowing this age group is the most fragile population against the virus can make them more worried about visiting the ED (93.27% of all COVID-19 deaths were aged 50 and above) [9].

CVA reduction in visitations (18.5% reduction, p-value 0.312) was not statistically significant, possibly due to the objective presentation of strokes (such as sudden unilateral weakness, slurred speech, and facial deviation). Patients and families of this population can see the effects of this condition, likely preventing the delay in the presentation of CVA, unlike ACS, where the main complaint is usually chest pain which is very much subjective.

DKA had the slightest reduction in the number of visitations (16% reduction, p-value 0.508). The mean age of DKA in our study was 22-24 years old, and the COVID-19 death percentage among ages <24 years is low [10], this can make parents less worried about visiting the ED.

In addition, blood glucose can be monitored at home using a glucometer, allowing the patients to recognize the severity of the disease.

All three conditions showed a reduction in visitations regardless of their statistical significance; delay of presentation of these conditions could lead to devastating effects on morbidity and mortality of these populations.

In a prospective observational study across three tertiary healthcare centers located in Saudi Arabia and Bahrain, they found a 50.49% decline in the number of ED visits of non-COVID-19-related emergencies during the lockdown period, with more than half the patients having a delayed presentation of more than 24 hours which was associated with a significantly higher rate of mortality. The study did not specify the indications for those ED visits. They attributed their findings to fear and curfew restrictions [8].

In a large retrospective multicentric American study, it was demonstrated that ED visits for serious cardiovascular conditions, including NSTEMI, ischemic stroke, hemorrhagic stroke, and heart failure, had declined significantly, reaching a nadir of 63% of the volume of the 2019 pre-pandemic period, with NSTEMI visits dropping the most, likely due to the varying and sometimes vague clinical presentation. Except for STEMI visits, the evidence for the decline was mixed, maintaining almost the same ratio as the pre-pandemic period [10].

On the contrary, a retrospective multicenter Finnish study exhibits a steady rate of ED visits for acute myocardial infarctions and strokes across the study period but a decrease of 11% for other heart diseases [11].

There are multiple ways to fight a pandemic, and one of them is forcing a nationwide lockdown; as we learned, it is a very effective temporary measure to control the spread of the virus as our analysis of Saudi Arabian statistics revealed COVID-19 mortality rates in the three months during and after lockdown of 0.8% and 1.8%, respectively [12].

A lockdown can be used to control the spread of an organism until it is understood; in terms of the nature of the virus, how it spreads, its severity, and long-term complications. Restriction of people's movement could negatively impact individuals and society (accessibility of healthcare system, economy, mental health).

COVID-19 was devastating and put every healthcare system to the extreme; decision-making and strategies to limit its harmful effect differed from country to country, which was reflected in their mortality rates. A future pandemic is not an unlikely event, and trying to prevent one may not be feasible; revision of current protocols and strategies to fight future pandemics and disasters could minimize the harm that comes with a pandemic.

Limitation

There are multiple limitations in our study. First, data were collected from a single center; consequently, our results may only represent a portion of Saudi Arabia ED visitations. Second, collecting data relied on ICD-10 codes; differences in using them may exist. Third, seasonal variation was not considered, as the periods compared were from the same year. Fourth, ACS and CVA were not subclassified due to a limited set of data. Fifth, we did not study the mortality rates nor the outcomes of these conditions before and during the lockdown. Sixth, the primary participants are coming to the ED, and other patients could have sought help through other sources, such as family medicine and telemedicine.

## Conclusions

A lockdown may be necessary to control a pandemic. However, it may carry potential collateral damage, such as a decrease and delay in the presentation of life-threatening conditions, which may lead to worsening outcomes.

A clinical presentation of these conditions should warrant comprehensive evaluation by healthcare workers regardless of an ongoing pandemic while implementing infection control guidelines.
